# Clinical Characteristics of Resected Acinar Cell Carcinoma of the Pancreas: A Korean Multi-Institutional Study

**DOI:** 10.3390/cancers13205095

**Published:** 2021-10-12

**Authors:** Sang Hyun Shin, Ho Kyoung Hwang, Jin-Young Jang, Hongbeom Kim, Sang Jae Park, Sung-Sik Han, In Woong Han, Dae Wook Hwang, Jin Seok Heo

**Affiliations:** 1 Division of Hepatobiliary-Pancreatic Surgery, Department of Surgery, Samsung Medical Center, Sungkyunkwan University School of Medicine, Seoul 06351, Korea; surgeonssh@skku.edu (S.H.S.); iw.han@samsung.com (I.W.H.); 2 Department of Hepatobiliary and Pancreatic Surgery, Yonsei University College of Medicine, Seoul 03722, Korea; drhhk@yuhs.ac; 3 Department of Surgery, Seoul National University Hospital, Seoul National University College of Medicine, Seoul 03080, Korea; jangjy4@snu.ac.kr (J.-Y.J.); surgeonkhb@snu.ac.kr (H.K.); 4 Department of Surgery, Center for Liver and Pancreatobiliary Cancer, National Cancer Center, Goyang 10408, Korea; spark@ncc.re.kr (S.J.P.); sshan@ncc.re.kr (S.-S.H.); 5 Division of Hepato-Biliary and Pancreatic Surgery, Department of Surgery, Asan Medical Center, University of Ulsan College of Medicine, Seoul 05505, Korea

**Keywords:** pancreatic acinar cell carcinoma, acinar cell carcinoma, pancreatic cancer

## Abstract

**Simple Summary:**

Pancreatic acinar cell carcinoma accounts for less than 1% of primary pancreatic neoplasms. Because of its rarity, its characteristics and clinical outcomes remain unclear. Treatment strategies for pancreatic acinar cell carcinoma have relied on those of pancreatic ductal adenocarcinoma. In previous studies, it has been difficult to identify its characteristics due to the lack of cohort numbers in single institutional studies with detailed data and the lack of detailed data in large cohort multi-institutional studies. This retrospective multicenter cohort used a database founded in 2015 by the Korean Association of Hepato-Biliary Pancreatic Surgery. This database has collected nationwide patient data with details. In the present study, we aimed to better understand clinical outcomes of resected pancreatic acinar cell carcinoma and to lay the groundwork for establishing proper treatment strategies.

**Abstract:**

Given the rare incidence of pancreatic acinar cell carcinoma (PACC), its post-resection clinical outcomes remain unclear. Treatment strategies for PACC have relied on those of pancreatic ductal adenocarcinoma (PDAC). The present study retrospectively investigated clinicopathologic characteristics of resected PACC registered in the Korea Tumor Registry System Biliary Pancreas database. Among 59 patients with a mean age of 59.2 years and a male predominance (83.1%), 43, 5, 7, and 4 had pure PACC, ductal differentiations, mixed neuroendocrine carcinomas, and intraductal and papillary variants, respectively. The mean tumor size was 4.6 cm, consisting of eight at T1, 26 at T2, and 25 at T3 stages. Metastasis to regional lymph node was identified in 15 (25.4%) patients. Thirty-one (52.5%) patients received adjuvant therapy. Five-year survival rate was 57.4%. The median survival was 78.8 months. In survival comparison according to the stage with AJCC system, N stage (lymph node metastasis), but not T stage, showed significant differences (*p* = 0.027). Resected PACC appeared to have clinical outcomes distinct from those of PDAC in this nationwide study. Therefore, large-scale multinational studies are needed to overcome the rarity of PACC and to establish an appropriate treatment strategies and staging system.

## 1. Introduction

The pancreas is an organ with exocrine and endocrine functions. The cells that occupy most of the exocrine cells are acinar cells that can secrete pancreatic enzymes [1]. Nevertheless, most malignant tumors originating from the exocrine pancreas are pancreatic ductal adenocarcinoma (PDAC), with pancreatic acinar cell carcinoma (PACC) accounting for less than 1% of primary pancreatic neoplasms [2,3,4]. Because of the rarity of PACC, it is difficult to know the exact characteristics of this disease and clinical course, as most studies reported to date only have limited numbers with data from single-center studies or multicenter studies [5,6,7,8,9,10].

It has been revealed that PACC is larger than PDAC at the time of diagnosis, with much better prognosis than PDAC [7,8,9,11,12,13]. However, its clinical outcome after surgical resection remains unknown. Studies on the role of adjuvant therapy and appropriate regimen for PACC are also insufficient. In American Joint Committee on Cancer (AJCC) staging, the staging of PACC is not differentiated from that of PDAC, but it is classified as an exocrine pancreatic cancer [14]. Although adjuvant therapy would offer the potential for reducing the risk of recurrence, a clear benefit of using adjuvant therapy has not been shown yet. Previous studies on adjuvant strategies for PACC have shown mixed results regarding their efficacies [6,8,15,16], which have mostly relied on studies of PDAC.

PACC is clearly a disease originating from cells different from PDAC, which requires independent research. Therefore, the purpose of the present study was to better understand the clinical outcome of resected PACC using the Korea Tumor Registry System Biliary Pancreas (KOTUS-BP), a Korean nationwide database, and to lay the groundwork for establishing proper treatment strategies.

## 2. Materials and Methods

### 2.1. Patients Database

This retrospective multicenter cohort study used a database founded in 2015 by the Korean Association of Hepato-Biliary Pancreatic Surgery (KAHBPS). This database, called KOTUS-BP, collected nationwide patient data retrospectively from institutions of KAHBPS members. There were 59 patients with biopsy-proven PACC resected with curative intention from the following seven institutions between January 2003 and December 2018: Severance Hospital, Ewha Womans University Hospital, Seoul National University Hospital, Seoul National University Bundang Hospital, Asan Medical Center, National Cancer Center, and Samsung Medical Center. Their clinicopathological, surgical, and postoperative data were reviewed, and their survival data were updated by each institution. This study was approved by our Institutional Review Board (IRB) (approval number: 2021-09-222). The requirement for written informed consent from participants was waived by our IRB due to the study’s retrospective nature.

Tumor, node, and metastasis (TNM) staging were classified according to the AJCC Cancer Staging Manual, 8th edition [14]. Resection margin status was categorized as R0 or R1. If the closest safe resection margin was less than 1 mm, it was categorized as an R1 margin [17,18]. Overall survival was measured as the period between surgery and death or the last visit to the outpatient center.

### 2.2. Statistical Analysis

All statistical analyses were performed using IBM SPSS 24 (SPSS Inc., Chicago, IL, USA). Probability (*p*) values of 0.05 or less were considered statistically significant. Actuarial survival and comparison of univariable analyses were performed using the Kaplan–Meier method with the log-rank test. Multivariable analysis was conducted using a Cox proportional hazard model to identify factors affecting patients’ overall survival.

## 3. Results

### 3.1. Patients’ Characteristics

Patients’ characteristics are summarized in Table 1. Of a total of 59 patients, there was a male predominance (83.1%). The mean age at diagnosis was 59.2 years. Preoperative CEA and CA19-9 levels were elevated in two (3.4%) and seven (11.9%) patients, respectively. The most predominant surgery was pancreaticoduodenectomy/pylorus-preserving pancreaticoduodenectomy (PD/PPPD) (*n* = 30, 50.8%), followed by distal pancreatectomy (DP) (*n* = 21, 35.6%). The mean tumor size at surgery was 4.6 cm. Historical variants of PACC were observed in 16 (27.1%) patients, including ductal differentiation in 5 (8.5%), mixed acinar-neuroendocrine carcinoma in 7 (11.9%), and intraductal and papillary variants in 4 (6.8%). According to cancer staging of AJCC 8th edition, 8 (13.6%), 26 (44.1%), and 25 (42.4%) patients had T1, T2, and T3 tumors, respectively. Lymph node metastasis was found in 15 (25.4%), consisting of 11 (18.6%) of N1 and 4 (6.8%) of N2. Complete microscopic resection (R0) was achieved in 55 (93.2%). In terms of adjuvant therapy, 31 (52.5%) patients underwent chemotherapy (*n* = 22, 37.3%) or concurrent chemo-radiation therapy (*n* = 9, 15.3%).

### 3.2. Treatment Outcomes and Prognostic Factors

**Figure 1:**
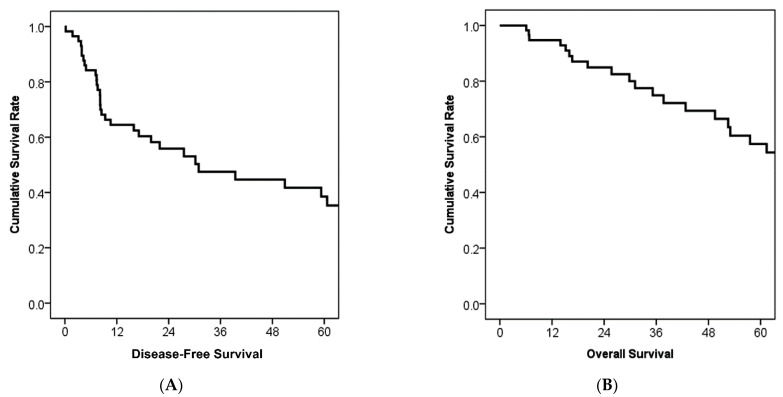
Kaplan–Meier survival curves of disease-free survival and overall survival (*n* = 59). (**A**) The median disease-free survival was 30.9 months and the 5-year disease-free survival rate was 38.5%. (**B**) The median overall survival was 78.8 months and the 5-year survival rate was 57.4%.

**Figure 2:**
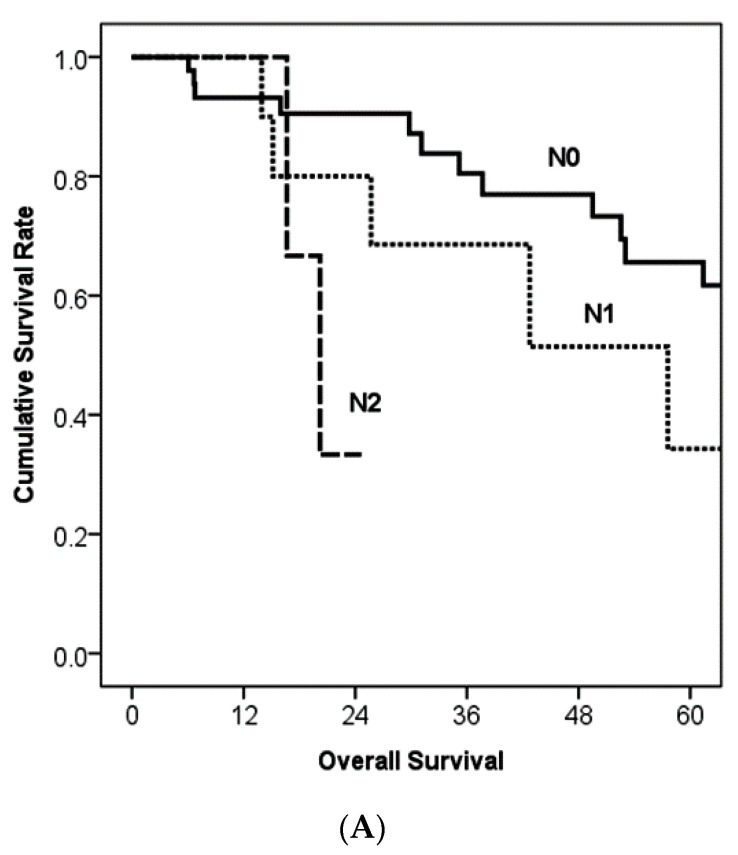

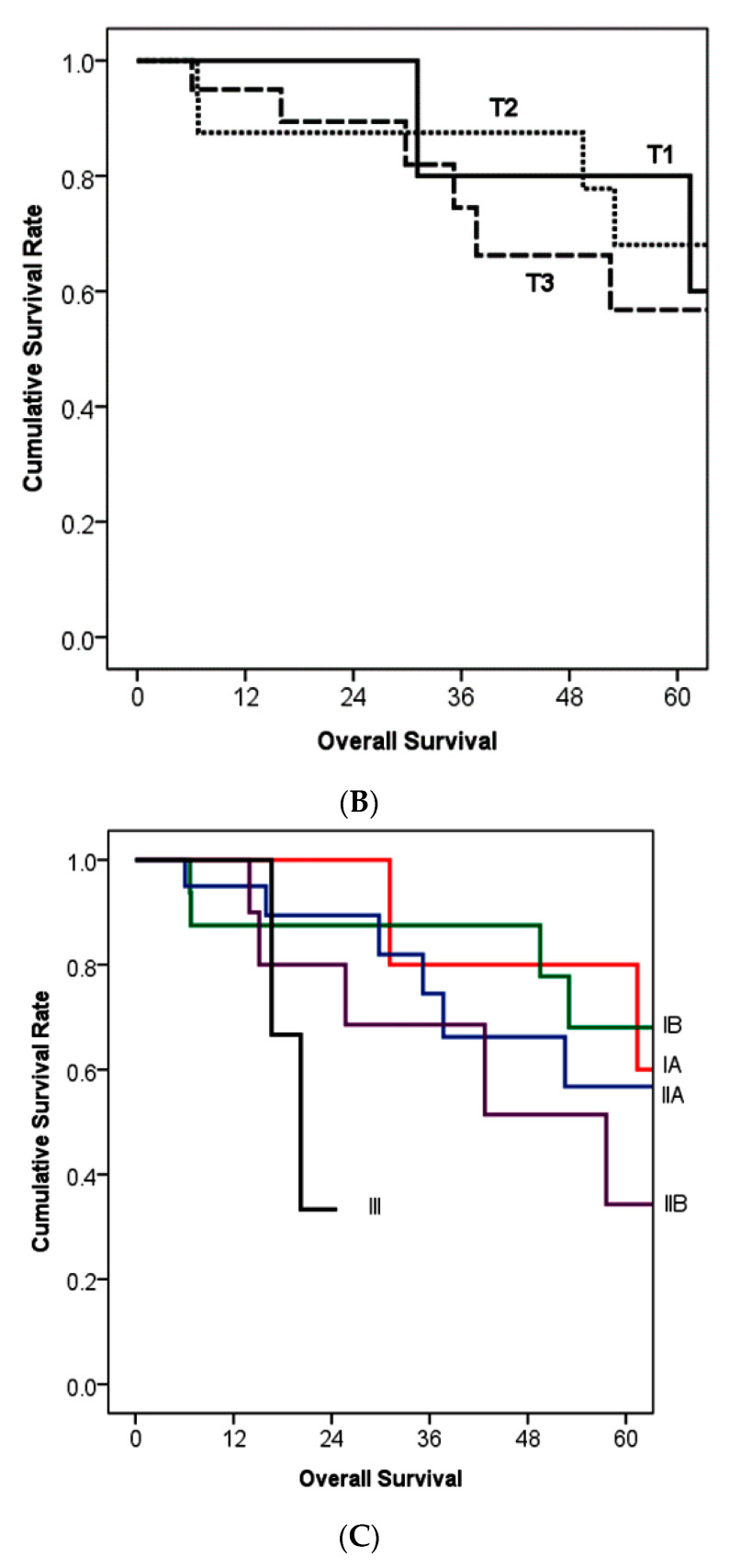
Kaplan–Meier survival curves according to the American Joint Committee on Cancer staging [14]. (**A**) When the analysis considered N stage (*n* = 59), there was a significant difference between N0 and N2 (*p* = 0.011), although there was no significant difference in comparison of N0 vs. N1 (*p* = 0.246) or N1 vs. N2 (*p* = 0.219). The median survival was 96.3 months in N0, 57.6 months in N1, and 20.2 months in N2. (**B**) When the analysis considered T stage in N0 patients (*n* = 44), there was no significant difference between T stages (*p* = 0.604 for T1 vs. T2, *p* = 0.263 for T2 vs. T3, and *p* = 0.729 for T1 vs. T3). The median survival was 77.5 months in T1, not reached in T2, and 78.8 months in T3. (**C**) The median survival was 77.4 months in stage IA, not reached in stage IB, 78.8 months in stage IIA, 57.6 months in stage IIB, and 20.2 months in stage III. Stage III showed significant differences with other stages (*p* = 0.21 for IA vs. III and *p* = 0.39 for IIA vs. III).

With a median follow-up period of 30.3 months, the median disease-free survival was 30.9 months, and the median overall survival was 78.8 months (Figure 1). Five-year disease-free survival and overall survival rates were 38.5% and 57.4%, respectively (Figure 1). Figure 2 shows survival comparisons according to cancer staging of the exocrine pancreatic tumor of the AJCC 8th edition. While N2 regional lymph node metastasis showed poorer prognosis than N0 (*p* = 0.011), N0 and N1 did not reflect survival differences (Figure 2A). In patients without regional lymph node metastasis (N0), T stage did not show survival differences between each stage (Figure 2B). The median survival was 77.4 months in stage IA, not reached in stage IB, 78.8 months in stage IIA, 57.6 months in stage IIB, and 20.2 months in stage III (Figure 2C).

**Figure 3:**
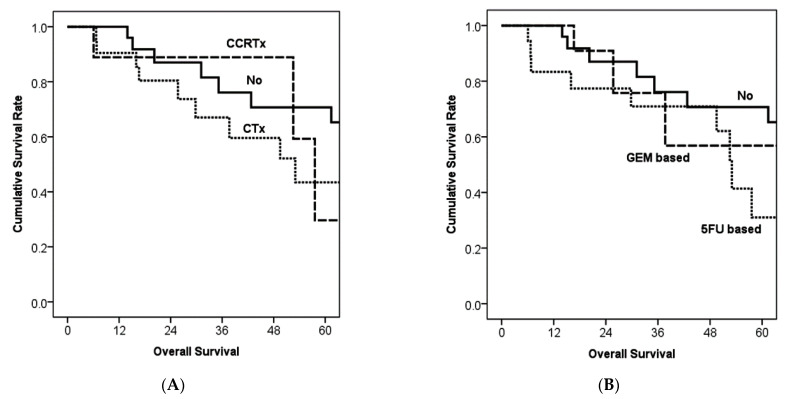
Kaplan–Meier survival curves according to the adjuvant therapy and its regimen. (**A**) Modalities of adjuvant therapy did not show significant survival differences. *p* value was 0.115 between no therapy and chemotherapy, 0.929 between chemotherapy and concurrent chemo-radiation therapy, and 0.199 between no therapy and concurrent chemo-radiation therapy. (**B**) In comparisons according to chemotherapy regimens, 5-fluorouracil-based chemotherapy showed better survival than no therapy (*p* = 0.035). However, gemcitabine-based chemotherapy did not show a significant survival difference compared with no therapy (*p* = 0.518) or compared with 5-fluorouracil-based chemotherapy (*p* = 0.196).

In terms of adjuvant therapy, the kind of therapeutic modality did not show significant survival differences (Figure 3A). Although gemcitabine (GEM)-based chemotherapy did not show significant survival difference compared with no therapy (*p* = 0.518) or compared with 5-fluorouracil (5-FU)-based chemotherapy (*p* = 0.196), 5-fluorouracil-based chemotherapy showed poorer survival than no adjuvant therapy (*p* = 0.035, Figure 3B).

Multivariable analysis using a Cox proportional hazard model (Table 2) revealed that patients’ overall survival was poor if they had elevated CA19-9 level (hazard ratio (HR): 24,078), N2 stage (HR: 13,882), and 5-fluorouracil-based chemotherapy (HR: 5733). On the other hand, an intraductal and papillary variant was associated with better survival (HR: 0.018).

## 4. Discussion

The present study revealed the characteristics and postoperative outcomes of resected PACC using a multi-institutional database of Korea. Compared with PDAC, PACC was detected at a younger age. In addition, tumor markers including CEA and CA19-9 did not seem to be sensitive markers in terms of clinical features of PACC. In terms of pathological features, PACC had a relatively larger size and lower rate of regional lymph node metastases than PDAC. TNM staging of exocrine pancreatic tumor of AJCC could not discriminate survival difference in PACC. In addition, PACC had better survival than PDAC, although current adjuvant therapy did not show a clear survival-improving effect.

In terms of tumor markers, elevated level of serum CA19-9 has been accepted as a sensitive marker for diagnosis of pancreatic cancer, with a pooled sensitivity of 75.4% in a previous meta-analysis [19]. Although CA19-9 can be elevated in nonmalignant diseases and other gastrointestinal tumors, it is elevated in approximately 65% of patients with pancreatic cancer. It has been the most widely accepted marker and prognostic factor to date for evaluating the performance of a treatment as well as for the diagnosis of initial or recurrent pancreatic cancer [20,21,22,23,24,25,26,27]. However, it is limited to studies on PDAC. In the present study, CA19-9 was elevated in only 11.9% of patients with PACC, similar to results of a previous study [9]. Although its elevation was associated with poorer survival (HR: 24,078, 95% CI: 2137–271,319), PACCs with normal CA19-9 levels accounted for a much larger proportion. Its prognostic value should be re-assessed in a larger cohort.

In most previous studies on PDAC, the mean tumor size at surgery is approximately 3 cm, and regional lymph node metastases are found for more than half of resected PDAC [25]. These two factors are always considered as prognosticators. Therefore, TNM cancer staging of exocrine pancreatic tumor in AJCC 8th edition was established for tumor size and number of regional lymph node metastasis. The present study showed that tumor size (T stage) did not reflect survival differences in resected PACC. Although lymph node metastasis (N stage) was significantly associated with survival, the rate of metastatic lymph node was low. It seemed difficult to adequately reflect the survival difference of resected PACC.

In terms of adjuvant therapy for resected pancreatic cancer, 5-FU- or GEM-based chemotherapy has been the mainstream regimen [28,29,30,31,32]. Although there has been no innovative medicine yet, various therapeutic strategies for adjuvant therapy have been recommended for patients with “pancreatic ductal adenocarcinoma”, showing effective benefit for survival. Although similar strategies have been applied to PACC in many institutes, the efficacy of adjuvant therapy has shown mixed results in previous studies [33,34]. Prospective randomized trials are still lacking. In the present study, 52.7% of all cohorts received adjuvant therapy including 5-FU- or GEM-based chemotherapy. Survival comparison showed that neither the therapeutic regimen nor the adjuvant therapy itself contributed to survival improvement. Rather, multivariable analysis showed poor survival in patients receiving 5-FU-based chemotherapy, which was thought to be due to the small number of study cohorts.

Although the present study was conducted using a nationwide multi-institutional database, there are limitations related to its retrospective design and the rarity of the disease. Patient cohorts of this study were inconsistent, including inaccurate diagnosis preoperatively and inconsistent postoperative treatment between institutions due to the lack of a unified therapeutic strategy. For example, since the adjuvant therapeutic strategies including chemotherapeutic regimen or radiotherapy were applied quite differently depending on the institution or physician, the exact chemotherapeutic regimen or radiotherapeutic dose could not be clearly presented in this study. Because of such inconsistency, there might be a limitation in understanding the natural history of resected PACC or the exact role of adjuvant therapy. In addition, despite a nationwide database being used to overcome the rarity, it still seemed difficult to completely overcome the numerical limitations of this rare disease. However, the present study was a study using the database of KAHBPS (KOTUS-BP), which contained detailed data from multi-institutions across the country to overcome the numerical limitations of a single institution [9,15] and the lack of detailed data of large cohorts [11,13] shown in previous studies.

## 5. Conclusions

In conclusion, although it was impossible to determine definite characteristics of PACC from this nationwide study, it confirmed that PACC and PDAC were clearly different diseases. The staging system designed based on the study of PDAC does not reflect the prognosis of PACC, and it is uncertain whether adjuvant therapeutic strategies targeting PDAC improve the prognosis of PACC. Further research is needed to establish through international collaboration proper treatment strategies for PACC.

## Figures and Tables

**Table 1:** Characteristics of patients (*n* = 59).

Characteristics	*n* (%) or Mean (±SD)	Characteristics	*n* (%) or Mean (±SD)
Age, year	59.2 (±11.6)	T stage, AJCC 8th edition	-
BMI, kg/m^2^	23.3 (±3.3)	T1	8 (13.6)
Sex	-	T2	26 (44.1)
Male	49 (83.1)	T3	25 (42.4)
Female	10 (16.9)	N stage, AJCC 8th edition	-
CEA	-	N0	44 (74.6)
Normal	55 (93.2)	N1	11 (18.6)
Elevated	2 (3.4)	N2	4 (6.8)
NA	2 (3.4)	Staging, AJCC 8th edition	-
CA19-9	-	Stage IA	8 (13.6)
Normal	47 (79.7)	Stage IB	16 (27.1)
Elevated	7 (11.9)	Stage IIA	20 (33.9)
NA	5 (8.5)	Stage IIB	11 (18.6)
Surgery	-	Stage III	4 (6.8)
PD/PPPD	30 (50.8)	R status	-
DP	21 (35.6)	R0	55 (93.2)
TP	3 (5.1)	R1	4 (6.8)
CP	1 (1.7)	Adjuvant therapy	-
Enucleation	4 (6.8)	No	28 (47.5)
Tumor location	-	Chemotherapy	22 (37.3)
Head	33 (55.9)	Chemo-Radiation therapy	9 (15.3)
Body	3 (5.1)	Chemotherapy regimen	-
Tail	22 (37.3)	No	28 (47.5)
Diffuse	1 (1.7)	5-fluorouracil based	18 (30.5)
Size, cm	4.6 (3.0)	Gemcitabine based	13 (22.0)
Pathology	-	-	-
Acinar cell carcinoma	43 (72.9)	-	-
Ductal differentiation	5 (8.5)	-	-
Neuroendocrine mixed	7 (11.9)	-	-
Intraductal and papillary variant	4 (6.8)	-	-

SD = standard deviation, BMI = body mass index, NA = not available, PD/PPPD = pancreaticoduodenectomy/pylorus-preserving pancreaticoduodenectomy, DP = distal pancreatectomy, TP = total pancreatectomy, CP = central pancreatectomy, AJCC = American Joint Committee on Cancer.

**Table 2:** Survival analysis for identifying factors affecting patients’ overall survival using a Cox proportional hazard model.

Factors	Univariable		Multivariable		
	Median Survival (Months)	*p*	*p*	Hazard Ratio	95% CI
Elevated CA 19-9	52.5	0.045	0.01	24.078	2137–271,319
Intraductal and papillary variant	Not reached	0.023	0.014	0.018	0.001–0.445
N2 stage	20.2	0.036	0.027	13.882	1339–143,931
5-fluorouracil-based	53.0	0.042	0.048	5.733	1015–32,379

CI = confidence interval.

## Data Availability

The datasets generated and/or analyzed during the current study are not publicly available but are available from the corresponding author on reasonable request.
